# The Current Status of the Soybean-*Soybean Mosaic Virus* (SMV) Pathosystem

**DOI:** 10.3389/fmicb.2016.01906

**Published:** 2016-11-30

**Authors:** Jian-Zhong Liu, Yuan Fang, Hongxi Pang

**Affiliations:** ^1^College of Chemistry and Life Sciences, Zhejiang Normal UniversityJinhua, China; ^2^College of Agronomy, Northwest A&F UniversityYangling, China

**Keywords:** soybean, *soybean mosaic virus*, disease resistance, virus-induced gene silencing, SNP, mapping

## Abstract

*Soybean mosaic virus* (SMV) is one of the most devastating pathogens that cost huge economic losses in soybean production worldwide. Due to the duplicated genome, clustered and highly homologous nature of *R* genes, as well as recalcitrant to transformation, soybean disease resistance studies is largely lagging compared with other diploid crops. In this review, we focus on the major advances that have been made in identifying both the virulence/avirulence factors of SMV and mapping of SMV resistant genes in soybean. In addition, we review the progress in dissecting the SMV resistant signaling pathways in soybean, with a special focus on the studies using virus-induced gene silencing. The soybean genome has been fully sequenced, and the increasingly saturated SNP markers have been identified. With these resources available together with the newly developed genome editing tools, and more efficient soybean transformation system, cloning SMV resistant genes, and ultimately generating cultivars with a broader spectrum resistance to SMV are becoming more realistic than ever.

## Overview

**FIGURE 1:**
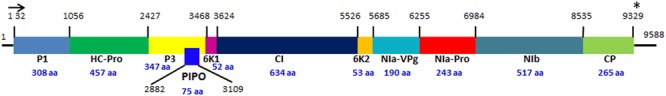
**The genome organization of *Soybean mosaic virus*.** The diagram was drawn based on the nucleotide sequence of SMV N strain (Eggenberger et al., 1989). The colored boxes represent 11 proteins encoded by SMV genome. The black lines at the 5′ and 3′ ends represent 5′ and 3′ untranslated region (UTR). The horizontal arrow and the star indicate the start and stop codons of the SMV polypeptide, respectively. The numbers above the vertical lines indicate the start positions of the SMV proteins. The sizes of the SMV proteins (the numbers of amino acids) are indicated by the blue numbers below the protein names. The PIPO embedded in the P3 is shown by the overlapping dark blue box with the start and stop positions labeled, respectively. The diagram is not drawn in scale.

Soybean [*Glycine max* L. (Merrill)] is one of the most important sources of edible oil and proteins. Pathogen infections cause annual yield loss of $4 billion dollars in the United States alone^1^. Among these pathogens, *Soybean mosaic virus* (SMV) is the most prevalent and destructive viral pathogen in soybean production worldwide (Hill and Whitham, 2014). SMV is a member of the genus *Potyvirus* in the *Potyviridae* family and its genome is a single-stranded positive-sense RNA, encoding at least 11 proteins (**Figure 1**): potyvirus 1 (P1), helper-component proteinase (HC-Pro), potyvirus 3 (P3), PIPO, 6 kinase 1(6K1), cylindrical inclusion (CI), 6 kinase 2 (6K2), nuclear inclusion a-viral protein genome-linked (NIa-VPg), nuclear inclusion a-protease (NIa-Pro), nuclear inclusion b (Nib), and coat protein (CP) (Eggenberger et al., 1989; Jayaram et al., 1992; Wen and Hajimorad, 2010). Numerous SMV isolates have been classified into seven distinct strains (G1 to G7) in the United States based on their differential responses on susceptible and resistant soybean cultivars (Cho and Goodman, 1979, **Table 1**), while in China, 21 strains (SC1–SC21) have been classified (Wang et al., 2003; Guo et al., 2005; Li et al., 2010). The relationship between G strains in the United States and SC strains in China has not been fully established yet. SMV resistance is conditioned by complex gene families. Multiple independent resistance loci with different SMV strain specificities have been identified, and most of them are non-Toll interleukin receptor- nucleotide binding site-leucine rich repeat (TIR-NBS-LRR) type R genes (Hill and Whitham, 2014). So far, three independent loci, *Rsv1, Rsv3*, and *Rsv4* in the United States and many *Rsc* loci in China, have been reported for SMV resistance. However, none of these genes has been cloned and their identities remain to be revealed.

**Table 1:** Summary of soybean-SMV studies.

Resistant locus	Chromosome location	Type of resistance gene	Strain specificity	Avirulent factor(s)
Rsvl	13 (Yu et al., 1994; Gore et al., 2002)	NBS-LRR type (Yu et al., 1994, 1996; Khatabi et al., 2013; Yang et al., 2013)	Resistant to: Gl–G4 susceptable to: G5–G7 (Chen et al., 1991)	He-Pro and P3 (Eggenberger et al., 2008; Hajimorad et al., 2008)
				Cl (Chowda-Reddy et al., 2011b; Wen et al., 2011)
				P3 (Chowda-Reddy et al., 2011b)

Rsv3	14 (Jeong et al., 2002; Shi et al., 2008)	CC- NBS-LRR type (Suh et al., 2011)	Resistant to: G5–G7 susceptable to: G1–G4 (Jeong et al., 2002)<	Cl (Seo et al., 2009; Zhang et al., 2009a; Chowda-Reddy et al., 2011b) P3 (Chowda-Reddy et al., 2011a,b)

Rsv4	2 (Hayes et al., 2000; Saghai Maroof et al., 2010)	Novel class (Ilut et al., 2016)	Resistantto: Gl–G7 (Chen et al., 1993; Ma et al., 1995)	P3 (Chowda-Reddy et al., 2011a,b; Khatabi et al., 2012; Wang et al., 2015)

## Mapping of SMV Resistant Loci

### Complex Nature of *Rsv1* Loci in Soybean

*Rsv1* was originally identified in the soybean line PI 96983 (Kiihl and Hartwig, 1979), and it confers extreme resistance (ER) to SMV-G1 through G6 but not to SMV-G7 (Chen et al., 1991; Hajimorad and Hill, 2001; **Table 1**). Multiple *Rsv1* alleles including *Rsv1-y, Rsv1-m, Rsv1-t, Rsv1*-*k*, and *Rsv1-r* have been identified from different soybean cultivars with differential reactions to SMV G1–G7 strains (Chen et al., 2001). *Rsv1* was initially mapped to soybean linkage group F on chromosome 13 (Yu et al., 1994) and two classes of NBS-LRR sequences (classes b and j) were identified in this resistance gene cluster (Yu et al., 1996). A large family of homologous sequences of the class j Nucleotide biinding site-leucine rich repeat (NBS-LRRs) clustered at or near the *Rsv1* locus (Jeong et al., 2001; Gore et al., 2002; Peñuela et al., 2002). Six candidate genes (1eG30, 5gG3, 3gG2, 1eG15, 6gG9, and 1gG4) in PI96983 were mapped to a tightly clustered region near *Rsv1*, three of them (3gG2, 5gG3, and 6gG9) were completely cloned and sequenced (GenBank accession no. AY518517–AY518519). Among the three genes, 3gG2 was found to be a strong candidate for *Rsv1* (Hayes et al., 2004). When 3gG2, 5gG3, and 6gG93 were simultaneously silenced using *Bean pod mottle virus* -induced gene silencing (BPMV-VIGS), the *Rsv1*-mediated resistance was compromised, confirming that one or more of these three genes is indeed the *Rsv1* (Zhang et al., 2012). Because, the sequence identities of these three *R* genes are extremely high along the entire cDNAs, it is impossible to differentiate which one(s) is *Rsv1.*

Several studies indicate that two or more related non-TIR-NBS-LRR gene products are likely involved in the allelic response of several *Rsv1*-containing lines to SMV (Hayes et al., 2004; Wen et al., 2013; Yang et al., 2013). Wen et al. (2013) generated two soybean lines, L800 and L943, derived from crosses between PI96983 (*Rsv1*) and Lee68 (*rsv1*) with distinct recombination events within the *Rsv1* locus. The L800 line contains a single PI96983-derived member 3gG2, confers ER against SMV-N (an avirulent isolate of G2 strain). In contrast, the line L943 lacks 3gG2, but contains a suite of five other NBS-LRR genes allows limited replication of SMV-N at the inoculation site. Domain swapping experiments between SMV-N and SMV-G7/SMV-G7d demonstrate that at least two distinct resistance genes at the *Rsv1* locus, probably belonging to the NBS-LRR class, mediate recognition of HC-Pro and P3, respectively (Khatabi et al., 2013; Yang et al., 2013).

### *Rsv3* Is Most Likely a NBS-LRR Type Resistant Gene

*Rsv3* was originated from “L29,” a ‘Williams’ isoline derived from Hardee (Bernard et al., 1991; Gunduz et al., 2000). The diverse soybean cultivars carrying *Rsv3* alleles condition resistance to SMV G5 through G7, but not G1 through G4 (Jeong et al., 2002; **Table 1**). *Rsv3* locus was firstly mapped between markers A519F/R and M3Satt on MLG B2 (chromosome 14) by Jeong et al. (2002), and was subsequently mapped on MLG-B2 with a distance of 1.5 cM from Sat_424 and 2.0 cM from Satt726 (Shi et al., 2008). The 154 kbp interval encompassing *Rsv3* contains a family of closely related coiled-coil NBS-LRR (CC-NBS-LRR) genes, implying that the *Rsv3* gene most likely encodes a member of this gene family (Suh et al., 2011).

### *Rsv4* Likely Belongs to a Novel Class of Resistance Genes

*Rsv4* confer resistance to all 7 SMV strains (Chen et al., 1993; Ma et al., 1995). It was identified in soybean cultivars V94-5152 and mapped to a 0.4 cM interval between the proximal marker Rat2 and the distal marker S6ac, in a ∼94 kb haplotype block on soybean chromosome 2 (MLG D1b++W) (Hayes et al., 2000; Saghai Maroof et al., 2010; Ilut et al., 2016). A haplotype phylogenetic analysis of this region suggests that the *Rsv4* locus in *G. max* is recently introgressed from *G. soja* (Ilut et al., 2016). Interestingly, this interval did not contain any NB-LRR type *R* genes. Instead, several genes encoding predicted transcription factors and unknown proteins are present within the region, suggesting that *Rsv4* most likely belongs to a novel class of resistance gene (Hwang et al., 2006; Ilut et al., 2016).

## The Other SMV Resistant Genes

Many *Rsc* loci have been identified. The resistance genes *Rsc-8* and *Rsc-9*, which confer resistance to strains *SC-8* and *SC-9* respectively, have been mapped to the soybean chromosomes 2 (MLG D1b+W) (Wang et al., 2004). The interval of *Rsc-8* was estimated to be 200 kb and contains 17 putative genes and five of them, Glyma02g13310, 13320, 13400, 13460, and 13470 could be the candidates of *Rsc-8* based on their predicted functions and expression patterns (Wang et al., 2011). The *Rsc-15* resistant gene was mapped between Sat_213 and Sat_286 with distances of 8.0 and 6.6 cM to the respective flanking markers on chromosome 6 (Yang and Gai, 2011). The resistance gene *Rsc-7* in the soybean cultivar Kefeng No.1 was mapped to a 2.65 mega-base (Mb) region on soybean chromosome 2 (Fu et al., 2006) and was subsequently narrowed down to a 158 kilo-base (Kb) region (Yan et al., 2015). Within 15 candidate genes in the region, one NBS-LRR type gene (Glyma02g13600), one HSP40 gene (Glyma02g13520) and one serine carboxypeptidase-type gene (Glyma02g13620) could be the candidates for *Rsc-7*. The allelic relationship between the *Rsv* loci and the *Rsc* loci has yet to be determined.

Despite numerous efforts, none of the SMV resistant genes has been cloned and their identities remain to be identified. This reflects the complex nature of the resistant genes in palaeopolyploid soybean, in which 75% of the genes are present in multiple copies (Schmutz et al., 2010). This statement is reinforced by a recent finding that the soybean cyst nematode (SCN) resistance mediated by the *Rhg1* is conditioned by copy number variation of a 31-kilobase segment, in which three different novel genes are present (Cook et al., 2012). There are 1–3 copies of the 31-kilobase segment per haploid genome in susceptible varieties, but 10 tandem copies in resistant varieties (Cook et al., 2012). The presence of more copies of the 31-kb segment in resistant varieties increases the expressions of this set of the 3 genes and thus conferes the resistance (Cook et al., 2012, 2014).

## Identification of Avirulent Factors in Different Smv Strains that are Specifically Recognized By Different *Rsv* Gene Products

### Avirulent Factors for *Rsv1*

SMV isolates are classified into seven strains (G1–G7) based on phenotypic reactions on a set of differential soybean cultivars (Cho and Goodman, 1982). The modification of avirulence factors of plant viruses by one or more amino acid substitutions can convert avirulence to virulence on hosts containing resistance genes and therefore, can be used as an approach to determine the avirulence factor(s) of a specific resistant gene.

*Rsv1*, a single dominant resistance gene in soybean PI 96983 (*Rsv1*), confers ER against SMV-G1 through G6 but not to SMV-G7 (Chen et al., 1991; Hajimorad and Hill, 2001; **Table 1**). SMV-N (an avirulent isolate of strain G2) elicits ER whereas strain SMV-G7 provokes a lethal systemic hypersensitive response (LSHR) (Hajimorad et al., 2003; Hayes et al., 2004). SMV-G7d, an evolved variant of SMV-G7 from lab, induces systemic mosaic (Hajimorad et al., 2003). Serial passages of a large population of the progeny in PI 96983 resulted in emergence of a mutant population (vSMV-G7d), which can evade *Rsv1*-mediated recognition and the putative amino acid changes that potentially responsible for the mutant phenotype is initially tentatively narrowed down to HC-Pro, coat protein, PI proteinase or P3 (Hajimorad et al., 2003; Seo et al., 2011) and was later mapped to P3 through domain swapping between the pSMV-G7 and pSMV-G7d (Hajimorad et al., 2005). The amino acids 823, 953, and 1112 of the SMV-G7d are critical in evading of *Rsv1*-mediated recognition (Hajimorad et al., 2005, 2006). By generating a series of chimeras between SMV-G7 and SMV-N in combination with site-directed mutagenesis, Eggenberger et al. (2008) and Hajimorad et al. (2008) independently showed that gain of virulence on *Rsv1*-genotype soybean by an avirulent SMV strains requires concurrent mutations in both P3 and HC-Pro and HC-Pro complementation of P3 is essential for SMV virulence on *Rsv1*-genotype soybean (**Table 1**). A key virulence determinant of SMV on *Rsv1*-genotype soybeans that resides at polyprotein codon 947 overlaps both P3 and a PIPO-encoded codon. This raises the question of whether PIPO or P3 is the virulence factor. Wen et al. (2011) confirmed that amino acid changes in P3, and not the overlapping PIPO-encoded protein, which is embedded in the P3 cistron, determine virulence of SMV on *Rsv1*-genotype soybean. Chowda-Reddy et al. (2011b) constructed a chimeric infectious clone of G7, in which the N-ternimal part of CI was swapped with the corresponding part of G2. Compared with wildtype G7, this chimeric strain lost virulence on *Rsv1*-genotype plant but gained infectivity on *Rsv3*-genotype plant, indicating an essential role of CI for breaking down both *Rsv1*- and *Rsv3*-mediated resistance (Chowda-Reddy et al., 2011b). Together, it appears that P3, HC-Pro and possibly CI are virulent determinants for *Rsv1*-mediated resistance (**Table 1**).

### Avirulent Factors for *Rsv3*

It has been proven that cytoplasmic inclusion cistron (CI) of SMV serves as a virulence and symptom determinant on *Rsv3*-genotype soybean and a single amino acid substitution in CI was found to be responsible for gain or loss of elicitor function of CI (Seo et al., 2009; Zhang et al., 2009a). Analyses of the chimeras by exchanging fragments between avirulent SMV-G7 and the virulent SMV-N showed that both the N- and C-terminal regions of the CI cistron are required for *Rsv3*-mediated resistance and the N-terminal region of CI is also involved in severe symptom induction in soybean (Zhang et al., 2009a). In addition to CI, P3 has also been reported to play an essential role in virulence determination on *Rsv3*-mediated resistance (Chowda-Reddy et al., 2011a,b; **Table 1**).

### Avirulent Factor for *Rsv4*

Gain of virulence analysis on soybean genotypes containing *Rsv4* genes showed that virulence on *Rsv4* carrying cultivars was consistently associated with Q1033K and G1054R substitutions within P3 cistron, indicating that P3 is the SMV virulence determinant on *Rsv4* and one single nucleotide mutation in the P3 protein is sufficient to compromise its elicitor function (Chowda-Reddy et al., 2011b; Khatabi et al., 2012; Wang et al., 2015). However, the sites involved in the virulence of SMV on *Rsv4*-genotype soybean vary among strains (Wang et al., 2015).

It is clear now that P3 plays essential roles in virulence determination on *Rsv1, Rsv3*, and *Rsv4* resistant loci, while CI is required for virolence on *Rsv1* and *Rsv3* genotype soybean plants (Chowda-Reddy et al., 2011a,b). These results imply that avirulent proteins from SMV might interact with the soybean *R* gene products at a converged point. This evolved interactions sometimes could give SMV advantage in breaking resistance conferred by different SMV resistant genes simply by mutations within a single viral protein. On the other hand, since multiple proteins are involved in virulence on different resistant loci, concurrent mutantions in multiple proteins of SMV are required to evade the resistance conferred by different SMV resistant genes. The likelihood of such naturally occuurred concurrent mutations in different viral proteins is low. Therefore, integration of all three SMV resistant genes in a single elite soybean cultivar may provide long-lasting resistance to SMV in soybean breeding practice (Chowda-Reddy et al., 2011b).

## Gain of Virulence By SMV on a Resistant Soybean Genotype Results in Fitness Loss in a Previously Susceptible Soybean Genotype

It seems that it is a common phenomenon that gain of virulence mutation(s) by an avirulent SMV strain on a resistant genotype soybean is associated with a relative fitness loss (reduced pathogenicity or virulence) in a susceptible host (Khatabi et al., 2013; Wang and Hajimorad, 2016). The majority of experimentally evolved mutations that disrupt the avirulence functions of SMV-N on *Rsv1*-genotype soybean also results in mild symptoms and reduced virus accumulation, relative to parental SMV-N, in Williams82 (*rsv1*), demonstrating that gain of virulence by SMV on *Rsv1*-genotype soybean results in fitness loss in a previously susceptible soybean genotype, which is resulted from mutations in HC-Pro, and not in P3 (Khatabi et al., 2013; Wang and Hajimorad, 2016). It has been also demonstrated that gain of virulence mutation(s) by all avirulent viruses on *Rsv4*-genotype soybean is associated with a relative fitness penalty for gaining virulence by an avirulence strain (Wang and Hajimorad, 2016). Thus, it seems that there is a cost for gaining virulence by an avirulence strain.

## The Soybean Lines Carrying Multiple *Rsv* Genes Display Broader Spectrum of Resistance Against SMV

Soybean line PI486355 displays broad spectrum resistance to various strains of SMV. Through genetic studies, Ma et al. (1995) identified two independently inherited SMV resistant genes in PI486355. One of the genes allelic to the *Rsv1* locus (designated as *Rsv1-s*) has dosage effect: the homozygotes conferring resistance and the heterozygotes showing systemic necrosis to SMV-G7. The other gene, which is epistatic to the *Rsv1*, confers resistance to strains SMV-G1 through G7 and exhibits complete dominance over *Rsv1.* The presence of this gene in PI486355 inhibits the expression of the systemic necrosis conditioned by the *Rsv1* alleles.

Soybean cultivar Columbia is resistant to all known SMV strains G1-G7, except G4. Results from allelism tests demonstrate that two genes independent of the *Rsv1* locus are present in Columbia, with one allelic to *Rsv3* and the other allelic to none of the known *Rsv* genes (Ma et al., 2002). Plants carrying both genes were completely resistant to both G1 and G7, indicating that the two genes interact in a complementary fashion (Ma et al., 2002). The resistance conditioned by these two genes is allele dosage-dependent, plants heterozygous for either gene exhibiting systemic necrosis or late susceptibility.

Tousan 140 and Hourei, two soybean accessions from Japan, and J05, a accession from China, carry both *Rsv1* and *Rsv3* alleles and are resistant to SMV-G1 through G7 (Gunduz et al., 2002; Zheng et al., 2006; Shi et al., 2011).

These results indicate that integration of more than one *Rsv* genes into one cultivar can confers a broader spectrum of resistance against SMV. Therefore, pyramiding multiple *Rsv* genes in elite soybean cultivars could be one of the best approaches to generate durable SMV resistance with broader spectrum.

## The Host Factors that are Involved in SMV Resistance

### The Host Components in *R* Gene-Mediated Defense Responses are Conserved in *Rsv1*-Mediated ER Against SMV

The key components in *R* gene mediated disease resistant signaling pathway have been identified in model plant *Arabidopsis*, among which, RAR1 (*Required for Mla 12 Resistance*), SGT1(*Suppressor of G2 Allele of Skp1*) and HSP90 (*Heat Shock Protein 90*) are the most important ones (Belkhadir et al., 2004). Using BPMV-VIGS, it has been shown that *Rsv1*-mediated ER against SMV in soybean requires RAR1 and SGT1 but not GmHSP90, suggesting although soybean defense signaling pathways recruit structurally conserved components, they have distinct requirements for specific proteins (Fu et al., 2009). However, Zhang et al. (2012) showed that silencing *GmHSP90* using BPMV-VIGS compromised *Rsv1*-mediated resistance. In addition, silencing *GmEDR1* (*Enhanced Disease Resistance 1*), *GmEDS1* (*Enhanced Disease Susceptibility 1*), *GmHSP90, GmJAR1* (*Jasmonic Responsive 1*), *GmPAD4* (*Phytoalexin Deficient* 4), and two genes encoding *WRKY* transcription factors (WRKY6 and WRKY 30), all of which are involved in defense pathways in model plant *Arabidopsis, Rsv1*-mediated ER was also compromised (**Table 2**). These results suggest that the host components required for *R* gene-mediated resistant signaling pathways are conserved across plant species.

**Table 2:** Host factors participate in SMV resistance.

Host factors	Biological functions	Type of resistance	Positive or negative Roles	Reference
GmHSP90, GmRARl	Defense signaling	Rsvl-mediated	Positive	Fu et al., 2009;
GmSGTl, GmEDSl, GmEDRl, GmJARl,				Zhang et al., 2012
GmPAD4, GmWRKY6, GmWRKY30				
GmMPK4	Defense signaling	Basal	Negative	Liu et al., 2011
GmMPK6	Defense signaling	Basal	Positive/negative	Liu et al., 2014
GmHSP40.1	Co-chaperone	Basal	Positive	Liu and Whitham, 2013
GmPP2C	ABA signaling	Rsv3-mediated	Positive	Seo et al., 2014
GmAKT2	K^+^ channel	Basal	Positive	Zhou et al., 2014
GmCNXl	Moco biosynthesis	Basal	Positive	Zhou et al., 2015
GmelF5A	Translation initiation	flsi/3-mediated	Positive	Chen et al., 2016a
GmeEFla	Translation elongation	Basal	Negative	Luan et al., 2016
GmAGOl	Gene silencing	Silencing-mediated	Positive	Chen et al., 2015
GmSGS3	Gene silencing	Silencing-mediated	Positive	Chen et al., 2015

### Conserved but Divergent Roles of MAPK Signaling Pathway in SMV Resistance

Mitogen-activated protein kinase (MAPK) cascades play important roles in disease resistance (Meng and Zhang, 2013). The function of MAPK signaling pathways in disease resistance was investigated in soybean using BPMV-VIGS (Liu et al., 2011, 2014, 2015). Among the plants silenced for multiple genes in MAPK pathway, the plants silenced for the *GmMAPK4 and GmMAPK6* homologs displayed strong phenotypes of activated defense responses (Liu et al., 2011, 2014). Consistent with the activated defense response phenotypes, these plants were more resistant to SMV compared with vector control plants (Liu et al., 2011, 2014), indicating that both genes play critical negative roles in basal resistance or PAMP-triggered immunity (PTI) in soybean. The constitutively activated defense responses has been reported for *mpk4* mutant in *Arabidopsis* (Petersen et al., 2000) and the positive role of MPK6 in defense responses is well-documented (Meng and Zhang, 2013). However, the negative role of MAKP6 homologs has not been reported previously (Liu et al., 2014), indicating that both conserved and distinct functions of MAPK signaling pathways in immunity are observed between *Arabidopsis* and soybean.

### Identifications of the Other Host Factors that Play Critical Roles in SMV Resistance

Numerous host factors participate in defense responses in plants. Identification of these factors may facilitate rationale design of novel resistant strategies. Recently, it has been shown that silencing *GmHSP40.1*, a soybean nuclear-localized type-III DnaJ domain-containing HSP40, results in increased infectivity of SMV, indicating a positive role of GmHSP40.1 in basal resistance (Liu and Whitham, 2013). A subset of type 2C protein phophatase (PP2C) gene family, which participate ABA signaling pathway, is specifically up-regulated during *Rsv3*-mediated resistance (Seo et al., 2014). Synchronized overexpression of GmPP2C3a using SMV-G7H vector inhibits virus cell-to-cell movement mediated by callose deposition in an ABA signaling-dependent manner, indicating that GmPP2C3a functions as a key regulator of *Rsv3*-mediated resistance (Seo et al., 2014). An ortholog of *Arabidopsis* K^+^ weak channel encoding gene AKT2, was significantly induced by SMV inoculation in the SMV highly resistant genotype, but not in the susceptible genotype (Zhou et al., 2014). Overexpression of *GmAKT2* not only significantly increased K^+^ concentrations in young leaves but also significantly enhanced the resistance against SMV, indicating alteration of K^+^ transporter expression could be a novel molecular approach for enhancing SMV resistance in soybean (Zhou et al., 2014). Molybdenum cofactor (Moco) is required for the activities of Moco-dependant enzymes. Cofactor for nitrate reductase and xanthine dehydrogenase (Cnx1) is known to be involved in the biosynthesis of Moco in plants. Soybean plants transformed with *Cnx1* enhanced the enzyme activities of nitrate reductase (NR) and aldehydeoxidase (AO) and resulted in an enhanced resistance against various strains of SMV (Zhou et al., 2015). The differentially expressed genes in *Rsv1* genotype in response to G7 infection have been identified (Chen et al., 2016a). Knocking down one of the identified genes, the eukaryotic translation initiation factor 5A (*eIF5A*), diminished the LSHR and enhanced viral accumulation, suggesting an essential role of eIF5A in the *Rsv1*-mediated LSHR signaling pathway. Eukaryotic elongation factor 1A (eEF1A) is a well-known host factor in viral pathogenesis. Recently, Luan et al. (2016) showed that silencing *GmeEF1A* inhibits accumulation of SMV and P3 protein of SMV interacts with GmeEF1A to facilitate its nuclear localization and therefore, promotes SMV pathogenicity.

## Small RNA Pathways in SMV Resistance

### miRNAs Participate in SMV Resistance

Small RNAs play a fundamental role in anti-viral defense. Three miRNAs, miR160, miR393 and miR1510, which have been previously shown to be involved in disease resistance in other plant species, have been identified as SMV-inducible miRNAs through small RNA sequencing approach (Yin et al., 2013), implying that these three miRNAs might play roles in SMV resistance. Chen et al. (2015) recently showed that the expression of *miRNA168* gene is specifically highly induced only in G7-infected PI96983 (incompatible interaction) but not in G2- and G7-infected Williams 82 (compatible interactions). Overexpression of *miR168* results in cleavage of miR168-mediated *AGO1* mRNA and severely repression of AGO1 protein accumulation (Chen et al., 2015). Silencing *SGS3*, an essential component in RNA silencing, suppressed AGO1 siRNA, partially recovers the repressed AGO1 protein, and alleviates LSHR severity in G7-infected *Rsv1* soybean (Chen et al., 2015). These results strongly suggest that miRNA pathway is involved in G7 infection of *Rsv1* soybean, and LSHR is associated with repression of AGO1.

Chen et al. (2016b) recently performed small RNA (sRNA)-seq, degradome-seq and as well as a genome-wide transcriptome analysis to profile the global gene and miRNA expression in soybean in response to three different SMV isolates. The SMV responsive miRNAs and their potential cleavage targets were identified and subsequently validated by degradome-seq analysis, leading to the establishment of complex miRNA-mRNA regulatory networks. The information generated in this study provides insights into molecular interactions between SMV and soybean and offer candidate miRNAs and their targets for further elucidation of the SMV infection process.

### Improving SMV Resistance through Generating RNAi Transgenic Lines Targeted for SMV Genome

The multiple soybean cultivars transformed with an RNA interference (RNAi) construct targeted for SMV HC-Pro displayed a significantly enhanced resistance against SMV (Gao et al., 2015). Soybean plants transformed with a single RNAi construct expressing separate short hairpins or inverted repeat (IR) (150 bp) derived from three different viruses (SMV, Alfalfa mosaic virus, and Bean pod mottle virus) confer robust systemic resistance to these viruses (Zhang et al., 2011). This strategy makes it easy to incorporate additional short IRs in the transgene, thus expanding the spectrum of virus resistance. As the cases in the other plant species, these studies demonstrate that RNA silencing is obviously the most effective approach for SMV resistance.

### VIGS Is a Powerful Tool to Overcome Gene Redundancy in Soybean

*Bean pod mottle virus* -induced gene silencing system has been proven successful in gene function studies in soybean (Zhang et al., 2009b, 2010; Liu et al., 2015). There are four *GmMAPK4* homologs that can be divided into two paralogous groups (Liu et al., 2011). The sequence identities of ORFs within the groups are greater than 96%, whereas the identities between the groups are 88.7% (Liu et al., 2015). The BPMV-VIGS construct used for silencing *GmMAPK4* by Liu et al. (2011) actually can silence all four of the isoforms simultaneously. When only one parologous group was silenced by using construct targeted for the 3′ UTR (the sequence identity of the 3′ UTRs between the two parologous groups is less than 50%), the activated defense response was not observed, indicating that silencing the four *GmMAPK4* isoforms simultaneously is necessary for activating defense responses in soybean. Using the same approach, it has been differentiated that *GmSGT1-2* but not *GmSGT1-1* is required for the *Rsv1*-mediated ER against SMV (Fu et al., 2009). Thus, VIGS is currently the most powerful tool in overcoming the gene redundancy in soybean.

## Concluding Remarks

As none of the SMV resistant gene has been cloned so far, it is not possible to generating resistant soybean plants simply by transforming the resistant genes. In addition, due to the rapid evolution in avirulence/effector genes, the resistance conditioned by *R* genes will be overcome quickly (Choi et al., 2005; Gagarinova et al., 2008). Therefore, there is urgent need for a better solution in generating long-lasting SMV resistance with wide spectrums. As the first step, the identities of different *Rsv* genes need to be revealed and the key components in SMV resistant signaling pathway need to be identified. Cutting edge functional genomics tools and technologies have been proven successful in cloning of SCN resistant genes *Rhg4* (Liu et al., 2011). TILLING coupled with VIGS and RNA interference confirmed that a mutation in the *Rhg4*, a serine hydroxymethyltransferase (*SHMT*) gene, is responsible for *Rhg4* mediated resistance to SCN (Liu et al., 2011). VIGS has been proven useful in interrogating gene functions and can overcome gene redundancy in soybean (Liu et al., 2015). It has been shown recently that knocking out all three *TaMLO* homoeologs simultaneously in hexaploid bread wheat using TALEN and CRISPR-CAS9 resulted in heritable broad-spectrum resistance to powdery mildew (Wang et al., 2014). We believe that the same strategy can be applied to soybean in the near future. These new functional genomics approaches and genome editing tools will greatly facilitate the cloning of SMV resistant genes and elucidating the SMV resistant signaling pathways. Marker assisted selection (MAS) has become very useful in the effort of tagging genes for SMV resistance. Single nucleotide polymorphism (SNP) is a powerful tool in genome mapping, association studies, and cloning of important genes (Clevenger et al., 2015) and the increasingly saturated SNPs are being established in soybean (Wu et al., 2010; Lee et al., 2015). With all these tools and resources available, pyramiding multiple SMV resistance genes in elite soybean cultivars to generate durable resistance with broad spectrum is more realistic than ever.

## Author Contributions

J-ZL wrote most part of this manuscript and prepared the figure and tables. YF and HP helped to write part of this manuscript.

## Conflict of Interest Statement

The authors declare that the research was conducted in the absence of any commercial or financial relationships that could be construed as a potential conflict of interest.

## Footnotes

1 http://aes.missouri.edu/delta/research/soyloss.stm
